# Dermoscopic characteristics of congenital melanocytic nevi in a cohort study in southern Brazil^☆^

**DOI:** 10.1016/j.abd.2021.03.016

**Published:** 2022-07-22

**Authors:** Camila Roos Mariano da Rocha, Thais Corsetti Grazziotin, Renan Rangel Bonamigo

**Affiliations:** a Universidade Federal de Ciências da Saúde de Porto Alegre, Porto Alegre, RS, Brazil; b Escola de Medicina, Pontifícia Universidade Católica do Rio Grande do Sul, Porto Alegre, RS, Brazil; c Faculdade de Medicina, Universidade Federal do Rio Grande do Sul, Porto Alegre, RS, Brazil; d Dermatology Service, Irmandade Santa Casa de Misericórdia de Porto Alegre, Porto Alegre, RS, Brazil

Dear Editor,

Congenital melanocytic nevi (CMN) are benign proliferations of melanocytes present at birth or which appear during the first two years of life.^1^ They are classically classified according to their largest diameter, as small (less than 1.5 cm), medium (between 1.5‒20 cm), and large or giant (greater than 20 cm).^2^ Larger lesions are more frequently associated with the development of melanoma and have a higher risk of extracutaneous complications (neurocutaneous melanocytosis). Regarding malignancy, prospective studies have established that the overall incidence of melanomas in CMN is low (1%‒2%). However, this incidence varies greatly according to the phenotype severity.^3^, ^4^, ^5^

Digital dermoscopy is a noninvasive test. The knowledge of dermoscopic characteristics is important so that this method can be used for patient diagnosis, follow-up, and management. There are few studies evaluating the evolution of the dermoscopic pattern of these nevi over time and its association with clinical and epidemiological characteristics. There is no standardization regarding the ideal period between assessments.^4^, ^6^, ^7^, ^8^, ^9^ The aim of the present study was to assess the clinical and dermoscopic characteristics of patients with CMN, comparing the dermoscopic findings at two medical consultations.

A retrospective cohort study was carried out to analyze the medical and photographic records obtained with digital videodermoscopy (Fotofinder Systems, GmbH, Bad Birnbach, Germany) of patients with CMN referred to the Dermoscopy Sector of a reference service in southern Brazil between 2016 and 2018. Data were collected on the patients general health status, dermatological anamnesis, the evolution of the congenital lesions, and stored macroscopic and microscopic photographic records. The description of variables and possible associations between clinical and dermoscopic aspects were verified.

The results were presented as central tendency measures (mean and median) and variability (standard deviation and interquartile range), in addition to absolute and relative distribution (n, %). The symmetry of continuous distributions was evaluated using the Kolmogorov-Smirnov test; Pearson's chi-square test (χ^2^) was used for the bivariate analysis of the qualitative variables.

Dermoscopic data were analyzed by two experienced evaluators and were based on the analysis of patterns classified as reticular (Fig. 1), globular (Fig. 2), homogeneous, reticular-homogeneous, globular homogeneous, reticular-globular, and acral patterns (parallel ridges or furrows, fibrillar, lattice and homogeneous). The evaluation was performed in two ways: a descriptive analysis of dermoscopic data of all lesions (those with only one record and those with follow-up; n = 82) and a comparative analysis of dermoscopic data of lesions that were followed over time. (n = 70). Both authors analyzed all recorded lesions and the final diagnostic definitions were attained by consensus. McNemar’s test was used to compare the data. The present study was approved by the Research Ethics Committee of the institution.

**Figure 1:**
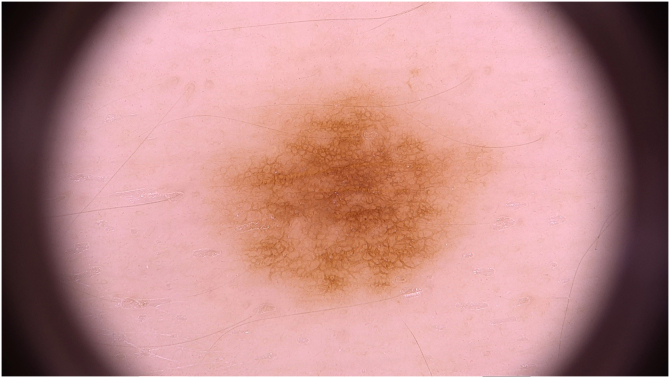
Brownish macula on dermoscopy showing a typical reticular pattern.

**Figure 2:**
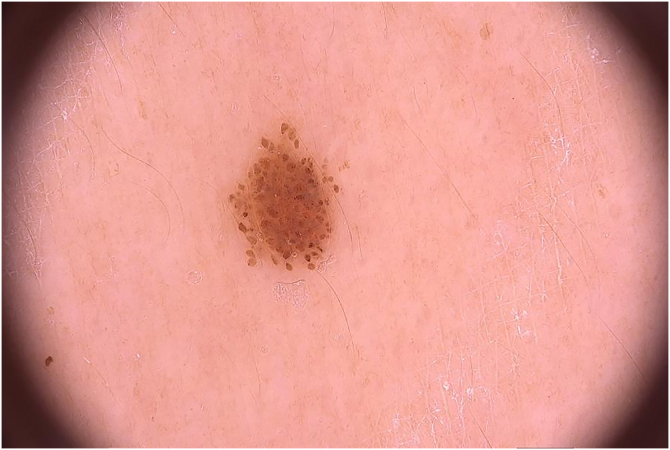
Small brownish papule on dermoscopy showing a typical globular pattern.

Eighty-two CMN were observed in 72 patients. Most individuals were phototype III (62.5%), had dark brown/black eyes (67.6%); medium/dark brown hair (38.9%) and 45.1% of the lesions were located on the extremities. Most lesions were small (58.5%; Table 1). The reticular pattern was the predominant dermoscopic pattern (31.7%), with the pigment network being the most common dermoscopic structure (70.7%; Table 2). The comparison between the first and the last clinical and dermoscopic examinations included 70 lesions, with a mean interval between them of 12.49 months and a median of 10 months (1^st^‒3^rd^ quartile: 8.0‒12.0), with the minimum period between the two assessments being 4 months and a maximum of 21 months. No morphological and structural differences were detected at the follow-up (p > 0.05; Table 3).

**Table 1:** Clinical and epidemiological characterization of patients with congenital melanocytic nevi (n = 72).

Variable	Total sample (n = 72)	
	n	%
**Sex**		
Male	30	41.7
Female	42	58.3
**Age (years)**		
Mean ± standard deviation (range)	27.2 ± 21.9 (1.0–76)	
Median (1^st^‒3^rd^quartile)	16.0 (10.3–43.7)	
**Phototype**		
II	6	8.3
III	45	62.5
IV	16	22.2
V	4	5.6
VI	1	1.4
**Eye color**		
Dark brown/black	48	67.6
Light brown/hazel	13	18.3
Light green	5	7.0
Blue	5	7.0
**Skin color/ethnicity**		
White	63	87.5
Brown	7	9.7
Black	2	2.8
**Hair color**		
Black	22	30.6
Dark/medium brown	28	38.9
Light brown	15	20.8
Blond	7	9.7
**Sunburns**		
Never	33	45.8
Once or more	39	54.2
**Use of sunscreen**		
Never	5	6.9
Occasionally	59	81.9
Always/daily	8	11.1
**Family history of skin cancer**		
No	54	75.0
Yes	18	25.0
**Number of CMN**		
More than one	9	12.5
One	63	87.5
**Location of the main CMN**		
Head and neck	7	8.5
Trunk	33	40.3
Extremities	37	45.1
Gluteal region	3	3.7
Inguinal region	2	2.4
**Classification of the main CMN**		
Small	48	58.5
Medium-sized	30	36.6
Large/Giant	4	4.9
**Recent modification**		
No	65	90.3
Yes	7	9.7
**Histopathological evaluation**		
No	68	94.4
Yes	4	0.6

**Table 2:** Dermoscopic pattern of congenital melanocytic nevi (n = 82).

Characteristics	Lesions assessed only once (n = 82)		Lesions with follow-up (n = 70)				
			Before		After		
	n	%	n	%	n	%	p^b^
**Dermoscopic pattern**							
Reticular	26	31.7	23	32.9	23	32.9	
Globular	14	17.1	12	17.1	12	17.1	>0.999
Reticulo-globular	12	14.6	9	12.9	9	12.9	
Homogeneous	17	20.7	15	21.4	15	21.4	
Reticular-homogeneous	12	14.6	11	15.7	11	15.7	
Parallel ridge	1	1.2					
**Color**							
Light brown	60	73.2	54	77.1	54	77.1	0.815
Dark brown	62	75.6	54	77.1	53	75.7	0.869
Black^a^	3	3.7	1	1.4	1	1.4	>0.999
Red^a^	4	4.9	3	4.3	3	4.3	>0.999
Blue^a^					1	1.4	>0.999
White^a^	3	3.7	2	2.9	2	2.9	>0.999
Blue-gray	7	8.5	6	8.6	6	8.6	>0.999
**Color symmetry**^a^					2		
Yes	77	93.9	65	95.6	65	95.6	>0.999
No	5	6.1	3	4.4	3	4.4	
**Symmetry of structures**							
Yes	76	92.7	66	94.3	66	94.3	>0.999
No	6	7.3	4	5.7	4	5.7	
**Pigment network**							
Yes	58	70.7	49	70.0	49	70.0	>0.999
No	24	29.3	21	30.0	21	30.0	
**Dots**							
Yes	49	59.8	41	58.6	41	58.6	>0.999
No	33	40.2	29	41.4	29	41.4	
**Globules**							
Yes	51	62.2	45	64.3	45	64.3	>0.999
No	31	37.8	25	35.7	25	35.7	
**Striae**							
No	82	100.0	70	100.0	70	100.0	‒
**Irregular striae**							
No	82	100.0	70	100.0	70	100.0	‒
**Structureless areas**							
Yes	9	11.0	6	8.6	6	8.6	>0.999
No	73	89.0	64	91.4	64	91.4	
**Regression**^a^							
Yes	1	1.2	1	1.4	1	1.4	>0.999
No	81	98.8	68	98.6	68	98.6	
**Hyperchromic macules**							
Yes	3	3.7	2	2.9	2	2.9	>0.999
No	79	96.3	68	97.1	68	97.1	
**Pseudocysts**							
Yes	5	6.1	5	7.1	5	7.1	>0.999
No	77	93.9	65	92.9	65	92.9	
**Perifollicular hyperpigmentation**^a^							
No	82	100.0	69	100.0	69	100.0	‒
**Perifollicular hypopigmentation**							
Yes	19	23.2	19	27.1	18	25.7	0.978
No	63	76.8	51	72.9	52	74.3	
**Hypertrichosis**							
Yes	19	23.2	15	21.4	14	20.0	>0.999
No	63	76.8	55	78.6	56	80.0	
**Blue-gray veil**							
No	82	100.0	70	100.0	70	100.0	‒
**Vascular structures**							
Yes	1	1.2	1	1.4	1	1.4	>0.999
No	81	98.8	69	98.6	69	98.6	
**Shiny white structures**^a^							
No	82	100.0	69	100.0	69	100.0	‒
**Negative pigment network**^a^							
Yes	4	4.9	4	5.8	4	5.8	>0.999
No	78	95.1	65	94.2	65	94.2	

a Missing data – Black color, Red color, blue color, white color, regression, perifollicular hyperpigmentation; shiny white structures, pigment network, atypical vascular pattern (1 [1.6%]); color symmetry, globules (2 [3,2]).

b McNemar-Bowker Test.

**Table 3:** Dermoscopic characteristics and follow-up interval.

Characteristics	Time of interval - months (n = 70)									
	Up to 10 months(n = 47)					>10 months (n = 23)				
	Before		After		p^b^	Before		After		p^b^
	n	%	n	%		n	%	n	%	
**Dermoscopic pattern**					‒					‒
Reticular	15	31.9	15	31.9		8	34.8	8	34.8	
Globular	9	19.1	9	19.1		3	13.0	3	13.0	
Reticulo-globular	5	10.6	5	10.6		4	17.4	4	17.4	
Homogeneous	8	17.0	8	17.0		7	30.4	7	30.4	
Reticular-homogeneous	10	21.3	10	21.3		1	4.3	1	4.3	
**Homogeneous pattern**										>0.999
Yes	19	40.4	19	40.4	‒	8	34.8	9	39.1	
No	28	59.6	28	59.6		15	65.2	14	60.9	
**Color symmetry**^a^					‒					‒
Yes	44	93.6	43	93.5		23	100.0	22	100.0	
No	3	6.4	3	6.5						
**Structure symmetry**					‒					‒
Yes	45	95.7	45	95.7		21	91.3	21	91.3	
No	2	4.3	2	4.3		2	8.7	2	8.7	
**Pigment network**					‒					‒
Yes	34	72.3	34	72.3		15	65.2	15	65.2	
No	13	27.7	13	27.7		8	34.8	8	34.8	
**Dots**					‒					‒
Yes	24	51.1	24	51.1		17	73.9	17	73.9	
No	23	48.9	23	48.9		6	26.1	6	26.1	
**Globules**					‒					‒
Yes	30	63.8	30	63.8		15	65.2	15	65.2	
No	17	36.2	17	36.2		8	34.8	8	34.8	

a Missing data – Pigment network, Color symmetry, globules (2 [3,2%]).

b McNemar-Bowker Test.

The lesions of four patients were submitted to histopathological analysis. The indications in these cases were as follows: the presence of a central papule in a medium-sized CMN located on the right shoulder of a 10-year-old patient, the presence of a central papule in a small lesion located on the fifth left toe of a 10-year-old patient, a satellite lesion in a patient with a large CMN located on the lumbar region and a lesion with structure asymmetry and a homogeneous pattern with predominant blue-gray color, located on the left thigh of a 10-year-old patient. All histopathological diagnoses were of compound melanocytic nevi. None of the assessed patients developed melanoma. Of the four patients with large/giant CMN included in the study, three had an indication for imaging examination and two had already undergone nuclear magnetic resonance with normal results.

Some studies have sought to describe specific dermoscopic features of CMN. Light brown globules with a central dot (target globules), target pigment network, focal pigment network thickening, perifollicular/focal hypopigmentation, and target vessels have been described as characteristics present in congenital lesions, although they were not specific. In the case of lesions that must be monitored over time, digital dermoscopy appears as an important tool. There are only a few studies describing the evolution and comparison of the dermoscopic features of these lesions and their correlations with the histopathological diagnoses.^10^ Regarding the clinical data of patients in the present study, it was observed that small nevi predominated, a result consistent with other published data.^8^, ^9^ The predominant dermoscopic pattern was the reticular one, regardless of the nevus location, unlike the data found in the literature, which found the globular pattern to be the most common. When comparing the predominant dermoscopic pattern with age, it was observed that the globular pattern was related to patients younger than 12 years, whereas the reticular pattern was related to those over this age, corroborating the data found in the literature.^10^, ^11^

A recent study found characteristics of atypical nevi present in CMN, such as radiating striae, focal hypopigmentation, atypical globules and blotches, regression, and blue-gray veil. In the present study, the authors did not find striae nor blue-gray veils; the vast majority of globules were typical, and regression was observed in only one lesion.^10^

In addition to the difficulty in the follow-up of patients, there is difficulty regarding the long follow-up time, necessary for any changes to appear. The present study sought to evaluate the dermoscopic characteristics and their changes during videodermoscopy follow-up and correlate them to biopsy indications and histopathological results. Using descriptive data, the clinical and dermoscopic characteristics of the CMN in the initial evaluation of the patients were demonstrated.

When comparing the images recorded in the initial and final evaluations, it was observed that there were no significant differences in the studied period. One of the limitations is the reduced follow-up time (mean of 12 months) since many of the expected outcomes (clinical and dermoscopic changes that indicate lesion excision, and the infrequent presence of malignancy) generally develop after many years. Another limitation is the diversity of the studied population, including children and adults, considering that lesions tend to be stable in the latter. The relatively small number of patients (72) is also be one of the limiting factors.

The present study obtained results that allow the clinical and dermoscopic characterization of patients with CMN, although studies with a larger sample size and a longer follow-up interval are necessary to demonstrate the real benefit of digital dermoscopy when monitoring these lesions over time.

## Financial support

None declared.

## Authors’ contributions

Camila Roos Mariano da Rocha: Statistical analysis; approval of the final version of the manuscript; design and planning of the study; drafting and editing of the manuscript; collection, analysis, and interpretation of data; intellectual participation in the propaedeutic and/or therapeutic conduct of the studied cases; critical review of the literature; critical review of the manuscript.

Thais Corsetti Grazziotin: Design and planning of the study; drafting and editing of the manuscript; collection, analysis, and interpretation of data; effective participation in research orientation; intellectual participation in the propaedeutic and/or therapeutic conduct of the studied cases; approval of the final version of the manuscript.

Renan Rangel Bonamigo: Statistical analysis; design and planning of the study; drafting and editing of the manuscript; collection, analysis, and interpretation of data; effective participation in research orientation; intellectual participation in the propaedeutic and/or therapeutic conduct of the studied cases; approval of the final version of the manuscript.

## Conflicts of interest

None declared.
